# Biodiesel production from microalgal isolates of southern Pakistan and quantification of FAMEs by GC-MS/MS analysis

**DOI:** 10.1186/1752-153X-6-149

**Published:** 2012-12-05

**Authors:** Syed Ghulam Musharraf, Muhammad Arif Ahmed, Noureen Zehra, Nurul Kabir, M Iqbal Choudhary, Atta-ur Rahman

**Affiliations:** 1 H.E.J. Research Institute of Chemistry, International Center for Chemical and Biological Sciences, University of Karachi, Karachi 75270, Pakistan; 2 Panjwani Center for Molecular Medicines and Drug Research, International Center for Chemical and Biological Sciences, University of Karachi, Karachi 75270, Pakistan; 3 Department of Biochemistry, Faculty of Science, King Abdulaziz University, Jeddah, 21412, Saudi Arabia

**Keywords:** Microalgae, Biodiesel algal resources of southern Pakistan, Fatty acid methyl ester, Gas chromatography, Tandem mass spectrometry

## Abstract

**Background:**

Microalgae have attracted major interest as a sustainable source for biodiesel production on commercial scale. This paper describes the screening of six microalgal species, *Scenedesmus quadricauda, Scenedesmus acuminatus, Nannochloropsis* sp*., Anabaena* sp*., Chlorella* sp*.* and *Oscillatoria* sp.*,* isolated from fresh and marine water resources of southern Pakistan for biodiesel production and the GC-MS/MS analysis of their fatty acid methyl esters (FAMEs).

**Results:**

Growth rate, biomass productivity and oil content of each algal species have been investigated under autotrophic condition. Biodiesel was produced from algal oil by acid catalyzed transesterification reaction and resulting fatty acid methyl esters (FAMEs) content was analyzed by GC/MS. Fatty acid profiling of the biodiesel, obtained from various microalgal oils showed high content of C-16:0, C-18:0, *cis*-Δ^9^C-18:1, *cis*-Δ^11^C-18:1 (except *Scenedesmus quadricauda*) and 10-hydroxyoctadecanoic (except *Scenedesmus acuminatus).* Absolute amount of C-14:0, C-16:0 and C-18:0 by a validated GC-MS/MS method were found to be 1.5-1.7, 15.0-42.5 and 4.2-18.4 mg/g, respectively, in biodiesel obtained from various microalgal oils. Biodiesel was also characterized in terms of cetane number, kinematic viscosity, density and higher heating value and compared with the standard values.

**Conclusion:**

Six microalgae of local origin were screened for biodiesel production. A method for absolute quantification of three important saturated fatty acid methyl esters (C-14, C-16 and C-18) by gas chromatography-tandem mass spectrometry (GC-MS/MS), using multiple reactions monitoring (MRM) mode, was employed for the identification and quantification of biodiesels obtained from various microalgal oils. The results suggested that locally found microalgae can be sustainably harvested for the production of biodiesel. This offers the tremendous economic opportunity for an energy-deficient nation.

## Background

Fossil fuel is now widely recognized as unsustainable resource with depleting supplies and increasing cost. Moreover, accumulation of carbon dioxide due to fossil fuels in the environment is getting higher. Therefore, renewable fuels are necessary for environmental and economic sustainability [1-3].

Biodiesel, as an alternative fuel, has attracted major interest worldwide in recent years. Conventional sources of biodiesel include plant oils and animal fats. The energy content, cetane number and viscosity of biodiesel are similar to those of petroleum based diesel fuel [4]. Chemically, biodiesel is a mixture of fatty acid mono alkyl esters. It is produced by a transesterification reaction between natural occurring triglycerides and alcohol in the presence of a catalyst [5]. Fatty acid methyl esters (FAMEs) have been quantified in various biodiesels by using several methods, including GC-MS [6,7] and LC-ELSD [8], HPTLC-EASI-MS [9] and GC-FID [10], while Guana et al. has recently reported a GC-MS/MS quantification of FAMEs in cyanobacteria [11].

Different raw materials have been explored for the biodiesel production, including edible plants/seeds such as canola, soybean, mustard, sunflower, palm oil, coconut and non-edible plants/seeds such as castor, pongame, jojoba, jatropa etc. [12]. However, plant resources have certain limitations i.e. competition with food, land utilization, long cultivation time, low yield, seed toxicity and only seeds contain oil for extractable quantities. Microalgae therefore, appear to be the cheapest source among all the renewable sources for the biodiesel production [13,14]. Oil productivity and growth rate of many microalgae greatly exceeds than the oil productivity from the best oil producing crops [13]. The average lipid content varies between 1 and 70% but under certain conditions some species can yield 80% of oil as their dry weight [14]. Most importantly microalgae do not compete with food crops in utilization, and can be produce in marginal lands, saline water bodies as well as in compact bioreactors.

There are about 50,000 microalgal species in the world in which about 30,000 have been explored for various purposes [15]. Pakistan possesses unique geological, geographical and atmospheric zones which support biodiversity. Algal flora is also abundant in Pakistan due to diverse water and rich saline land habitats. An important need is to explore this native floral wealth for biodiesel production. Fatty acid composition in various algal groups has been investigated in Pakistan [16,17]. However, Ahmad et al. have recently reported biodiesel production from mixed algal culture [18]. To the best of our knowledge, this is the first report describing the biodiesel production and its analysis from algal oil of purified microalgae species obtained from southern Pakistan. Similarly, a GC-MS/MS method for the quantification of tetradecanoic acid methyl ester (C-14:0), hexadecanoic acid methyl ester (C-16:0) and octadecanoic acid methyl ester (C-18:0) in various microalgal species is also reported here.

## Method

### Chemicals and reagents

FAME standards (RM-5 and Rapeseed mix) and BF_3_/methanol (derivitizing reagent) were purchased from Sigma-Aldrich (USA). Sodium hydroxide was purchased from Uni-Chem (England). Sulphuric acid (conc.) were purchased from Mecrk (Germany). Chemicals for algal media were purchased from BioM Laboratories (Cerritos, USA).

### Microalgae collection, isolation and identification

Microalgae were collected from both marine as well as fresh water habitats. Fresh water samples were collected from different places of Karachi (southern Pakistan), including Liyari River and Kalari Lake, whereas marine samples were collected from Hawksbay, Paradise Point, Buleji and Korangi creek. All samples were collected during June 2008 to May 2009. Various physical parameters were recorded at the collection spots, including pH, temperature and salinity of water. Moreover, the season of collection and the habitat of microalgae were also taken into account. Samples were collected in a number of falcon tubes containing different types of sterilized media.

Microalgae were grown in 250 mL Erlenmeyer flasks containing Bold’s basal medium for fresh water microalgae, whereas Guillard's f2 medium and enriched sea water medium were used for marine samples. Media were evenly distributed in flasks and autoclaved for inoculation of the microalgal seed cultures. Flasks were placed in a growth chamber assembled with 18 watt florescent light. Inoculated flasks were aerated with flow rate of 5 L per min. Microalgae were isolated by using micromanipulation, serial dilution and streak plating methods [19]. Compound microscope (BX60; Olympus Corp., LakeSuccess, NY) was used for preliminary observation, while Nikon TE-2000E microscope (Melville, NY) was used for detailed examination and imaging. Microalgal species were identified by the Miss Noureen Zehra using previous reported literature [20-24].

### Large scale cultivation of algae

Isolated microalgae were grown autotrophically in 5 L fermenter (Biostat Q, B. Braun, and Germany) for oil extraction. Actively growing starter cultures (100 mL) were inoculated into the fermenter, containing 5 L of autoclaved media. Cultures were grown under continuous illumination of florescent light at 27±0.5°C and aerated with air. The cell count of cultures was monitored daily using a haemocytometer (HBG-Germany). Microalgae were harvested for chemical analysis when the cultures attained the late-logarithmic phase. Culture medium was centrifuged at 4,000 rpm at 20°C for 10 min in a centrifuge machine (J-6 MI centrifuge, Beckman Coulter, USA). Pellets were then washed twice with 0.5 M ammonium formate to remove salt. The wet cell mass was frozen overnight at −70°C and then lyophilized in a freeze drier (Christ Alpha 1–2 LD Plus, Germany) to measure cell dry weight and total lipid content.

### Lipid extraction and derivitization of oilgae

Lipids were extracted according to Bligh and Dyer protocol [24]. The weight of lipids was measured after evaporating the solvent on a rotary evaporator (N-1000, Eyela, Japan). Percent lipid content was calculated by dividing the weight of these oils by dry weight of microalgae.

Transesterification was carried out by taking 25 mg of the algal oil sample and treating with 1.5 mL NaOH. The reaction mixture was stirred and heated at 100°C for 5 min. The reaction mixture was then cooled at room temperature before adding the esterfying agent. 2 mL of BF_3_/methanol reagent (14%) was added in the reaction mixture and it was heated at 100°C for 5 min. The reaction mixture was again cooled to 30–40°C, followed by the addition of 1 mL of isooctane and 5 mL of saturated NaCl solution and agitated thoroughly. The isooctane layer was separated from the aqueous layer into a clean glass tube and the whole extraction procedure was repeated again. Total 2 mL of isooctane was stored at 4°C till analysis.

### Characterization of biodiesel

Saponification and iodine values were determined according to the method of Gopinath et al. [25]. Following parameters were used to calculate saponification and iodine value. Saponification value (SV) = 268–(0.418 X P)–(1.30 X S)–(0.695 X O)–(0.77 x L)–(0.847 X LL) Iodine Value (IV) = 35.9–(0.212 X P)+(0.660 X S)+(0.448 X O)+(1.23 X L)+(1.73 X LL). Higher heating values of biodiesels were calculated according to Ayhan Demirbas model [26]. Higher heating value (HHV) = 49.43–(0.015 x IV)–(0.041 X SV) where, P = palmitic, S = stearic, O = oleic, L = linoleic and LL = linolenic. Cetane number, kinematic viscosity and density of biodiesel were calculated from the FAMEs composition of every oil according to the protocol of Ramírez-Verduzco et al. [27].

### GC/MS analysis

GC-MS Analysis of biodiesel produced from various microalgal oil was performed on Agilent 7000A Triple quadrupole mass spectrometer, coupled to a gas chromatograph (Agilent 7890) equipped with an auto sampler. The GC column used was a fused with silica capillary column (Agilent 190905–433, 30m × 250 μm i.d., film thickness 0.25 μm). The pressure of the carrier gas (helium) was 7.0699 Psi at the initial oven temperature with flow rate 64 mL min^−1^. All standards and samples were injected in the split mode (split/column flow ratio 60:1). The injector temperature was 250°C; the oven temperature was 50°C, rose to 220°C at rate of 14°C min^−1^ (total run time 34 min). The mass spectrometer was operated in the electron impact (EI) mode at 70 eV in the scan range of 50–650 *m/z*. The temperature of the transfer line and of the ion source was set to a value of 320 and 280°C, respectively. The injection sample volume was 1.0 μL. Mass Hunter software (Agilent) was used for data acquisition and processing. Peak identification of algal oil was performed by comparison with retention times of standards and the mass spectra obtained compared with those available in the Wiley and NIST libraries (Wiley Registry TM, 8th Edition Mass Spectral Library, and the NIST 08 Mass Spectral Library (NIST/EPA/NIH) 2008 version) with an acceptance criterion of a match above a critical factor of 80%.

Quantification of FAMEs (C-14:0, C-16:0 and C-18:0) was achieved in MRM mode with the same GC conditions with collision energy of 30 eV and a solvent delay of 5 min. The dwell time was 50 ms and the scan rate was 6.5 cycles/s. The fragment ions (listed in Table 1) allowed quantification by using one of the three ions as the quantification ion and the other two as qualifiers. Calibration standard solutions, with concentration ranging between 60 to 150 ng/μL, were prepared by appropriate dilution with isooctane in 5 mL volumetric flasks. Calibration curves were plotted by peak area analytes versus analytes concentration. In order to estimate the limits of detection (LOD) and quantitation (LOQ), a calibration curve was used. The LOD and LOQ were calculated according to the following equation: LOD = 3.3δ/S and LOQ = 10δ/S, where δ the residual standard deviation of a regression line or the standard deviation of Y-intercepts of regression line, S the slope of the calibration curve.

**Table 1:** Optimized GC-MS/MS acquisition method parameters for FAMEs precursor ions and product ions for qualitative and quantitative analysis

**FAMEs**	**Precursor ion (*m/z*)**	**Optimized collision energy (ev)**	**MRM transitions (*m/z*)**	
			**Identification**	**Quantification**
Tetradecanoic acid methyl ester	242.4	10	184.8; 157.1; 128.7	242.4>157.1
Hexadecanoic acid methyl ester	270.4	10	227.1; 199.0; 171.2	270.4>171.2
Octadecanoic acid methyl ester	298.5	10	255.1; 212.4; 101.0	298.5>101.0

## Results and discussion

30 Marine algal samples were collected from the different locations around the coast of Karachi, Pakistan and about 20 fresh water algal samples were collected from fresh water ponds and lake. The average temperature of the surface water was 25–29°C for both fresh and marine water samples. Salinity range was 0.5-1 ppt and 36–39 ppt for fresh and marine water samples, respectively. Similarly, pH range was 6.8-7.5 and 7.7-8.1 for fresh and marine water samples, respectively.

**Figure 1:**
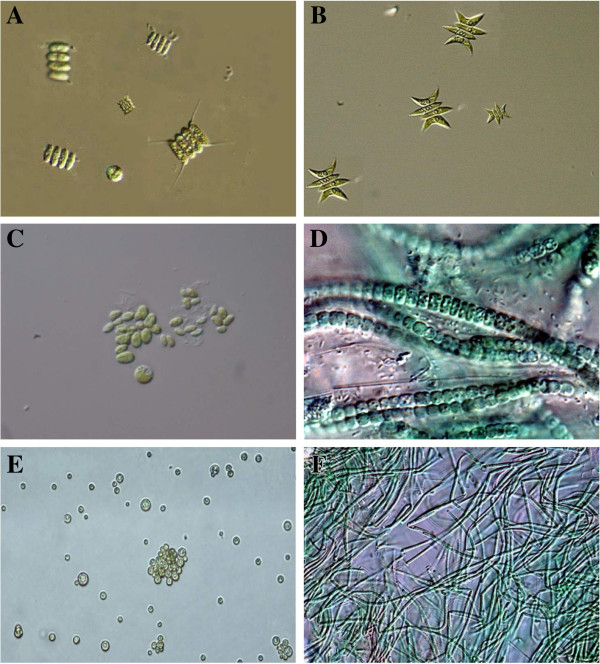
**Differential interference contrast (DIC) images of isolated unimicroalgal species (A) *Scenedesmus quadricauda *(B) *Scenedesmus acuminatus *(C) *Nannochloropsis *sp. (D) *Anabaena *sp. (E) *Chlorella *sp. and (F) *Oscillatoria *sp.**

Most of the microalgal samples were grown successfully in the laboratory and belongs to the class Chlorophyceae, Cyanophyceae and Bacillariophyceae (Diatoms). Six microalgal strains, KU-001*,* KU-002*,* KU-003*,* KU-004, KU-005, and KU-006 were successfully isolated by using microlgal isolation techniques [19] and identified as *Scenedesmus quadricauda, Scenedesmus acuminatus,Nannochloropsis* sp.*, Anabaena* sp.*, Chlorella* sp. and *Oscillatoria* sp. using their morphological features [20-23]. Strains KU-001*,* KU-002*,* KU 004*,* and KU 005 were obtained from fresh water while strains KU 003 and KU 006 were obtained from the marine samples. Microscopic images of purified algal strains are presented in Figure 1.

### Cell biomass production and comparison of lipid content

The purified strains were cultivated for the autotrophic biomass production. Biomass productivity rate and percent oil content of each microalgae are summarized in Table 2. *Oscillatroria* sp. has the highest biomass production rate (2166.7 mg/d/L) among all screened microalgal species. However, biomass productivity (under autotrophic condition) of commercially available *S. quadricauda* (190 mg/d/L)*, S. acuminatus* (210 mg/d/L), *Nannochloropsis* sp. (170–210 mg/d/L)*,* and *Chlorella* sp. (230 mg/d/L) have been investigated by Rodolfi et al. [28]. Microalgae biomass was harvested by centrifugation when growth of the microalgal species reached to its threshold value. The lipid content in all microalgal algal species was expressed in terms of percentage of their dry weight. *S. acuminatus*, *S. quadricauda* and *Nannochloropsis* sp. showed high oil percent i.e. 17.0, 12.6 and 10.4%, respectively, while *Anabaena* sp*.*, *Chlorella* sp*.* and *Oscillatroria* sp. showed oil content < 5.5%. Reported literature showed that *S. quadricauda*, *Nannochloropsis* sp., *Chlorella* sp. and *Oscillatroria* sp. contain 18.4, 29.2, 18.7 and 5.0% oil, respectively, in phototrophic cultivation without any stress [28,29]. This variation in biomass productivity and oil productivity may be due the temperature, pH, salinity and other climatic difference. Therefore, it is important to study native microalgal species for their biomass and oil productivity. Among all screened microalgal species, *S. acuminatus* is found to be the most promising oil producing species (17.0%) and it was found to be suitable for biodiesel production on a large scale whereas *Oscillatroria* sp. has shown highest biomass productivity rate (Table 2).

**Table 2:** Biomass and algal oil productivity of various microalgal species

**S. No.**	**Algal specie (Codes)**	**Habitat**	**Growth rate (mg/day/L)**	**Oil percent (%)**
1.	*S. quadricauda* (KU-001)	Fresh water	22.79	12.6
2.	*S. acuminatus* (KU-002)	Fresh water	94.74	17.0
3.	*Nannochloropsis* sp. (KU-003)	Marine	148	10.4
4.	*Anabaena* sp. (KU-004)	Fresh water	275.62	2.98
5.	*Chlorella* sp. (KU-005)	Fresh water	152.65	5.3
6.	*Oscillatroria* sp. (KU-006)	Marine	2166.7	3.69

### Characterization of biodiesel

**Figure 2:**
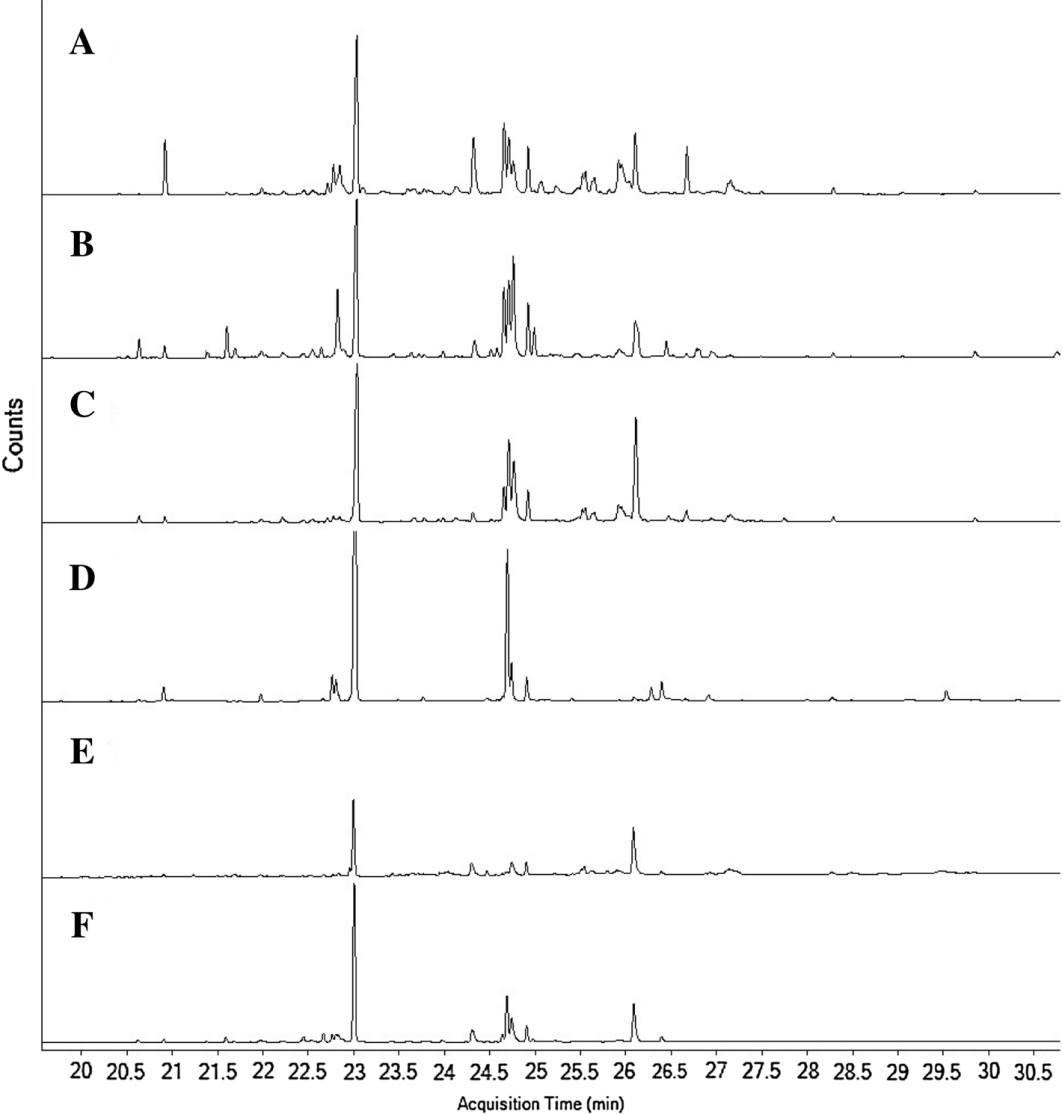
**Total ion chromatogram (TIC) of biodiesel synthesized from microalgal oil (A) *Anabaena *sp. (B) *Chlorella *sp. (C) *Nannochloropsis *sp. (D) *Scenedesmus acuminatus *(E) *Scenedesmus quadricauda *and (F) *Oscillatoria *sp.**

The suitable characteristics needed in algal species for optimum biodiesel production are high growth rate, high biomass concentration and high oil content. In addition to these, they must have the right kind of FAMEs content needed for a high quality biodiesel [30]. Characterization of fatty acid methyl esters of synthesized biodiesel was carried out by GC-MS. The comparative total-ion chromatogram of all the samples is shown in Figure 2. Identified peaks of the fatty acid methyl esters and their relative percentages are summarized in Table 3.

**Table 3:** FAMEs Profile of biodiesel obtained from various microalgal species by GC-MS

**RT**		**Microalgae**					
**(Min)**	**Label**	**Strain KU-001**	**Strain KU-002**	**Strain KU-003**	**Strain KU-004**	**Strain KU-005**	**Strain KU-006**
18.59	Dodecanoic acid methyl ester	-	-	-	-	0.68	-
20.90	Tetradecanoic acid methyl ester	1.40	1.91	0.78	7.65	1.58	0.73
22.63	7,10-Hexadecdienoic acid methyl ester	-	-	0.65	1.33	1.37	2.66
22.76	9-Hexadecenoic acid methyl ester	-	2.92	0.65	3.12	-	1.33
22.81	11-Hexadecenoic acid methyl ester	-	2.21	0.41	0.00	10.17	0.60
23.01	Hexadecanoic acid methyl ester	37.48	61.58	36.80	29.18	30.15	50.05
24.30	9(*R*),10(*R*)-Dihydroxy octadecanoic acid methyl ester	10.52	-	1.78	11.56	3.70	6.19
24.51	6,9,12-Octadecatrienoic acid methyl ester	-	-	-	-	1.12	-
24.64	9,12-Octadecadienoic acid methyl ester	-	-	4.19	8.92	8.69	1.28
24.69	9-Octadecenoic acid methyl ester	-	21.22	11.18	5.16	7.82	11.48
24.74	11-Octadecenoic acid methyl ester	9.31	3.31	8.99	2.41	11.34	6.17
24.91	Octadecanoic acid methyl ester	6.80	3.45	5.06	6.15	7.51	4.60
25.06	11-Methoxy octadecanoic acid methyl ester	-	-	-	2.63	-	-
25.46	8,11-Eicosadienoic acid methyl ester	-	-	-	0.49	-	-
25.54	11,14-Eicosadienoic acid methyl ester	-	-	0.82	1.13	-	-
25.91	8,9-Dihydroxy docosanoic acid methyl ester	-	-	1.18	2.14	1.38	-
26.08	10-Hydroxy octadecanoic acid methyl ester	34.49	-	22.20	9.15	9.68	14.94
26.39	9,10-Epoxy octadecanoic acid methyl ester	-	2.21	-	-	-	-
26.44	11-Eicosenoic acid methyl ester	-	-	0.59	-	2.16	-
26.66	Eicosanoic acid methyl ester	-	-	2.09	6.98	0.44	-
26.93	13,16-Docosadienoic methyl ester	-	1.20	-	0.35	0.34	-
28.27	Docosanoic acid methyl ester	-	-	0.84	0.95	0.73	-
29.84	Tetracosanoic acid methyl ester	-	-	0.72	0.70	1.14	-
31.84	Hexacosanoic acid methyl ester	-	-	1.08	-	-	-

**Figure 3:**
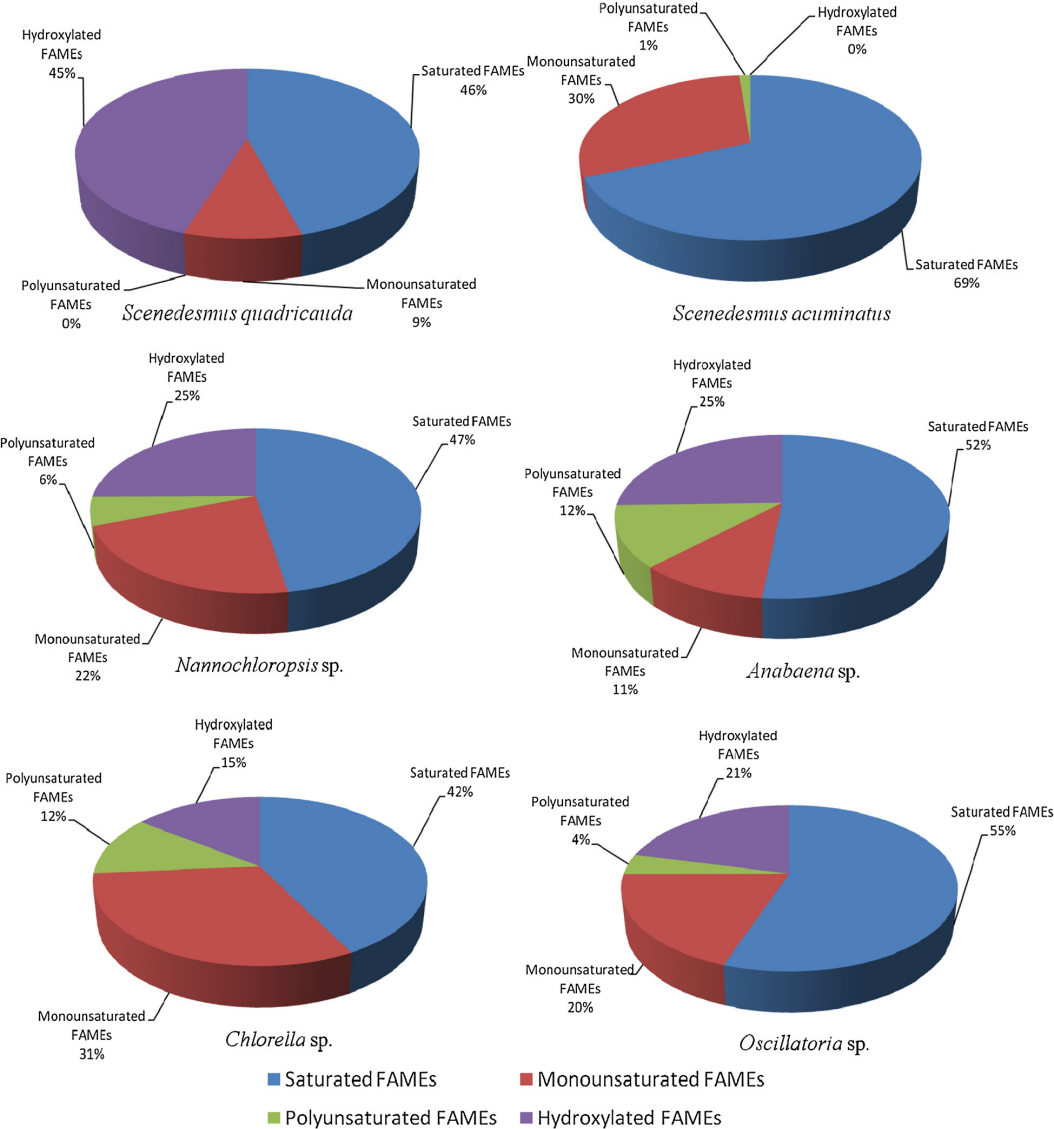
Distribution of FAMEs among various microagal strains.

It was observed that, *S. quadricauda*, *S. acuminatus, Nannochloropsis* sp., *Anabaena* sp., *Chlorella* sp. and *Oscillatroria* sp. contain total saturated fatty acid methyl esters (SAFA) of 46, 69, 47, 52, 42, and 55%, while the total monounsaturated (MUFA) were 9, 30, 22, 11, 31 and 20%, respectively. Polyunsaturated fatty acid methyl acid (PUFA) contents were found to be 0, 1, 6, 12, 12 and 4%, respectively as shown in Figure 3. Moreover, all the species were found rich in hexadecanoic acid (C-16:0) methyl ester, ranging from 29-61%. The fatty acid profiling of microalgae ultimately affects the quality of the biodiesel. The carbon chain length of saturated and unsaturated fatty acids affects biodiesel properties, such as cetane number, oxidative stability and cold-flow properties. Generally, high proportion of SAFAs and MUFAs are preferred for increasing energy yield and superior oxidative stability. However, oils containing MUFAs are prone to solidification at low temperatures, while oils rich in PUFAs have very good cold-flow properties, but such biodiesel tends to be vulnerable to oxidation. This tendency causes adverse effects on fuel conservation and combustion [31]. In strain KU-001 (*S. quadricauda*) no PUFA was observed. Strains KU-002 (*S. acuminatus*), KU-003 (*Nannochloropsis* sp.) and KU-005 (*Chlorella* sp.) are found to have high cetane number, as they are rich in octadecanoic acid methyl ester (C-18:0), oleic acid methyl ester (C-18:1) and linoleic acid methyl ester (C-18:2). Hydroxylated saturated fatty acid methyl esters were found in all microalgal samples in high percentages, ranging between 15-45% (except *S. acuminatus*). Density, kinematic viscosity, iodine value, higher heating value and centane number for all six microalgal biodiesels are summarized in the Table 4. All properties of the biodiesels are found within the range of ASTM standards, except for strain KU-001(*S. quadricauda*) which shows high cetane number.

**Table 4:** Properties of biodiesel obtained from various microalgae

**Properties**	**Strain KU-001**	**Strain KU-002**	**Strain KU-003**	**Strain KU-004**	**Strain KU-005**	**Strain KU-006**	**ASTM biodiesel standard**
Iodine value (gm/100 g of oil)	36.61	36.11	45.63	52.87	46.03	67.36	-
Saponification value (mg KOH/g of oil)	237.02	220.72	228.79	232.71	230.71	209.34	-
Density (gm/cm^3^)	0.476	0.838	0.622	0.647	0.642	0.874	0.82-0.90
Kinematic viscosity (mm^2^/s)	2.5	4.3	3.3	3.4	3.3	4.3	1.9-6.0
Higher heating value (MJ/Kg)	39.16	39.84	39.36	39.10	39.28	39.84	>35
Cetane number	40.0	67.5	49.6	50.3	49.8	63.6	Min. 47

### Quantification of FAMEs by GC-MS/MS

**Figure 4:**
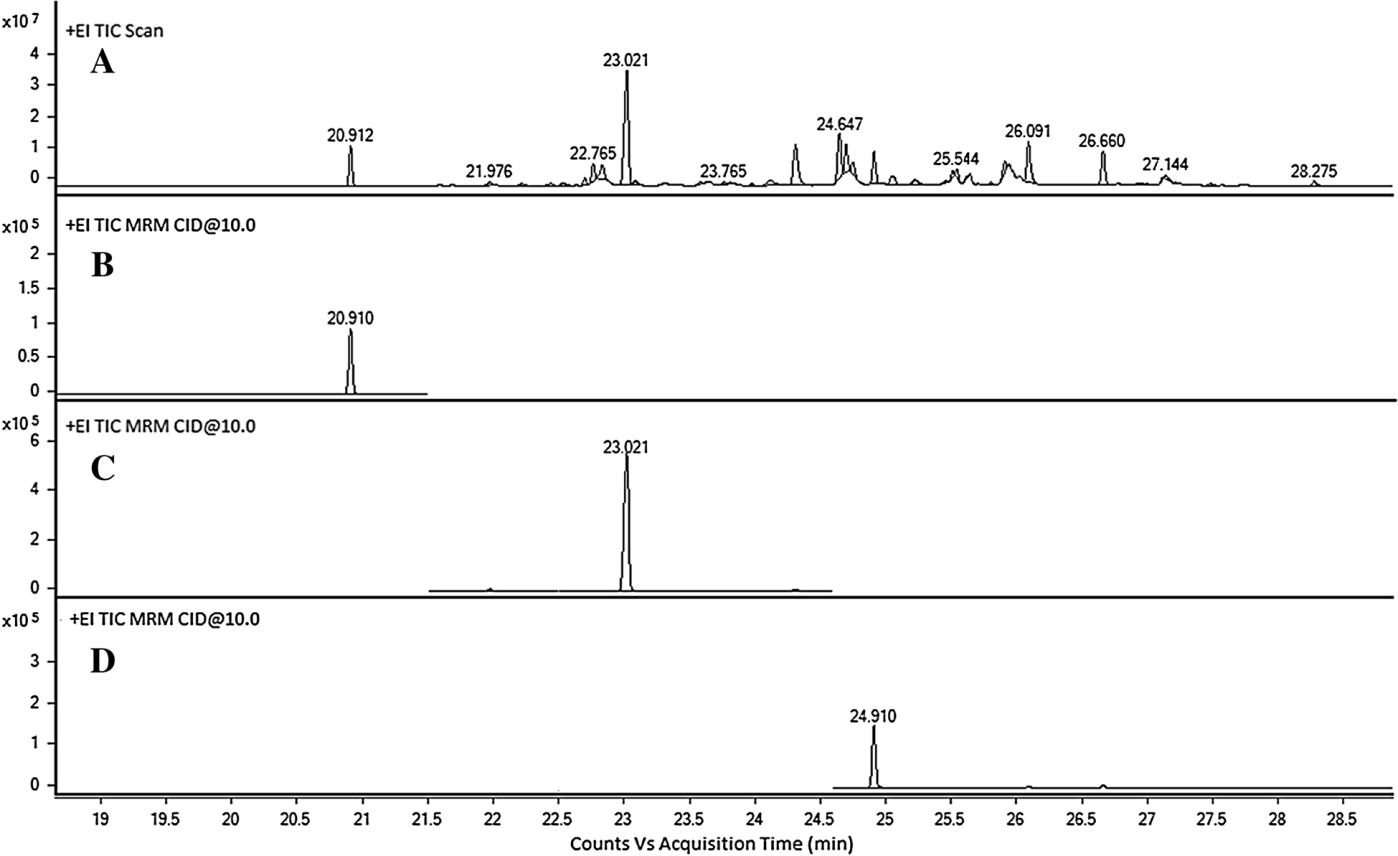
**(A) Full scan MS chromatogram of biodiesel synthesized from microalgal oil (B) Reconstructed ion chromatogram for tetradecanoic acid methyl ester at *m/z *242.4→72.7+100+157.1 (C) Reconstructed ion chromatogram for hexadecanoic acid methyl ester at *m/z *270.4→100+58.6+132 (D) Reconstructed ion chromatogram for octadecanoic acid methyl ester at *m/z *298.5 →72+101+100.8+198.**

Three biodiesel based fatty acid methyl esters i.e. C-14:0, C-16:0 and C-18:0 were investigated quantitatively by gas chromatography-tandem mass spectrometry in multiple reaction monitoring (MRM) mode (Figure 4). MRM scan mode has a higher selectivity than the SIM scan mode [32], and thus allows a better signal resolution without a preliminary fractionation of the oil. Only the molecules which have the selected fragmentation patterns are taken into account. Therefore, the interferences are dramatically reduced and the signal to noise ratio increases by many orders of magnitude. The first quadrupole isolates all the M^+·^ ions with a *m/z* 242.4, 270.4 and 298.5, while in the collision cell these ions undergo a fragmentation process which yield the formation of product ions. Collision energy (10 to 40 eV) was varied in the product ion scan and 10 eV was found to be the most suitable for obtaining fragment ions. Characteristic fragment ions for these three saturated fatty methyl esters are listed in Table 1. Out of three, singal best transition was selected for quantification and the other two were marked as qualifiers, as indicated in Table 1. In the third quadrupole, only the product ions were isolated and their ions were considered in the mass chromatogram. In this way, the interferences were eliminated from the chromatogram and only the ions with the selected precursor ion and product ions were taken into account.

The linearity, LOD and LOQ of biodiesel based saturated fatty acid, analyzed by GC-MS/MS were investigated using calibration samples, and the results are presented in Table 5. Linear relationships between the concentration and the corresponding peak area were found over the concentration range of 60–150 ng/μL with good correlation coefficient (r^2^) ≥099.2 in all cases. The LODs and LOQ varied between from 4.27-17.65 ng/μL and between 14.22-58.82 ng/μL, respectively. The validated GC-MS/MS method was applied to six samples of algal oil for the determination of absolute amount of biodiesel based fatty acid methyl esters, including C-14:0, C-16:0 and C-18:0, and the results are summarized in the Table 6. The amount of C-14:0 is found highest in strain KU-004 and lowest in strain KU-002. Strain KU-003 shows high amounts of both C-16:0 and C-18:0, whereas strain KU-004 and Strain KU-002 showed the lowest amounts of C-16:0 and C-18:0, respectively.

**Table 5:** Retention time, correlation coefficients, limits of detection, limits of quantification

**FAMEs**	**Retention time (min)**	**Correlation coefficient (r**^**2**^**)**	**Limits of detection (LOD) (ng/μL)**	**Limits of quantification (LOQ) (ng/μL)**
Tetradecanoic acid methyl ester	20.91	0.998	4.27	14.22
Hexadecanoic acid methyl ester	23.02	0.998	17.65	58.82
Octadecanoic acid methyl ester	24.91	0.992	17.20	57.33

**Table 6:** Absolute amount of fatty acid methyl ester analysis of oilgae

**Sample**	**FAMEs (mg/g of oil)**		
	**Tetradecanoic acid methyl ester**	**Hexadecanoic acid methyl ester**	**Octadecanoic acid methyl ester**
*S. quadricauda*	3.057±0.019	45.590±1.101	24.851±0.946
*S. acuminatus*	3.069±0.009	67.318±0.650	8.568±0.192
*Nannochloropsis* sp.	3.289±0.008	83.836±1.068	36.969±0.349
*Anabaena* sp.	6.006±0.053	30.465±0.465	21.191±0.166
*Chlorella* sp.	3.289±0.074	39.275±1.841	27.060±1.151
*Oscillatroria* sp.	3.176±0.002	66.111±1.440	23.256±0.215

## Conclusion

The study involved the screening of six microalgal strains, obtained from water bodies of southern Pakistan for the biodiesel production. *Scenedesmus acuminatus* contains the highest oil content among all six microalgal strains, whereas *Oscillatroria* sp. was found to have highest biomass productivity (per day per liter). Fatty acid profiling of the biodiesel produced from the microalgal oil shows high content of saturated and monounsaturated FAMEs, including of C-16:0, C-18:0, *cis*-Δ^9^C-18:0 and *cis*-Δ^11^C-18:0. Density, kinematic viscosity, iodine value, higher heating value and cetane number of the biodiesels were found to be within range. Moreover, the three most important saturated FAMEs i.e. C-14:0, C-16:0 and C-18:0 in microalgal biodiesels have been determined quantitatively by GC-MS/MS. Further efforts on screening more microalgae for biodiesel production are needed on discovering the best microalgae that would be feasible for biodiesel production in terms of biomass productivity and resulting oil content in local environment.

## Competing interests

The authors declare that they have no competing interests.

## Authors’ contributions

SGM: Participated in the experimental designing and method optimization. MAA: Participated in bench work and played a role in manuscript writing. NZ: Involved in microalgal culturing, isolation and identification. NK: Involved in the microscopy of microalgal species. MIC: Involved in the useful discussion critical analysis of results and manuscript writing. AR: Involved in the useful discussion and participated in manuscript writing. All authors read and approved the final manuscript.
